# Ozurdex® Implant Inside the Capsular Bag: A Report of a Rare Case

**DOI:** 10.7759/cureus.101142

**Published:** 2026-01-09

**Authors:** Lalit Verma, Bhanu Priya, Priksha Lakhlan, Bharat R Thoumungkan, Avnindra Gupta

**Affiliations:** 1 Ophthalmology/Vitreo-Retina Services, Centre for Sight, New Delhi, IND; 2 Ophthalmology/Strabismus, Cataract, and Refractive Surgery, Centre for Sight, New Delhi, IND

**Keywords:** anterior segment optical coherence tomography (as-oct), capsular bag, chronic uveitis, complicated cataract, ozurdex implant

## Abstract

We report a rare case of an intravitreal dexamethasone implant (Ozurdex®) inadvertently located within the capsular bag following a uneventful cataract surgery. A 46-year-old female patient with a history of recalcitrant uveitis in the left eye presented with diminished vision in the right eye. She was diagnosed with a complicated cataract with 360° posterior synechiae. After manual small incision cataract surgery and intraocular lens implantation, the patient received an intravitreal Ozurdex implant to mitigate possible exacerbation of preexisting uveitis. Postoperatively, the implant was visualized within the capsular bag, confirmed by anterior segment optical coherence tomography. This rare event highlights an unusual complication of intravitreal Ozurdex injection and emphasizes the importance of technique and anatomical considerations during administration.

## Introduction

The dexamethasone intravitreal implant, Ozurdex® (Allergan, Inc., Irvine, California, United States), is widely used in the management of macular edema secondary to uveitis, retinal vein occlusion, and diabetic macular edema [1]. The implant is designed to deliver sustained release of corticosteroid within the vitreous cavity via a biodegradable polymer matrix. Although Ozurdex has a well-established safety profile, several complications have been documented, including implant migration into the anterior chamber, sulcus, and subconjunctival space, particularly in eyes with posterior capsular defects or aphakia [2]. We report an extremely rare event where an Ozurdex implant was identified inside the capsular bag following cataract surgery, an occurrence not previously described in the literature, to our knowledge.

## Case presentation

A 46-year-old woman presented with complaints of progressive diminution of vision in her right eye. Her ocular history was significant for recalcitrant uveitis in the left eye, leading to complete vision loss five years earlier. 

**Figure 1:**
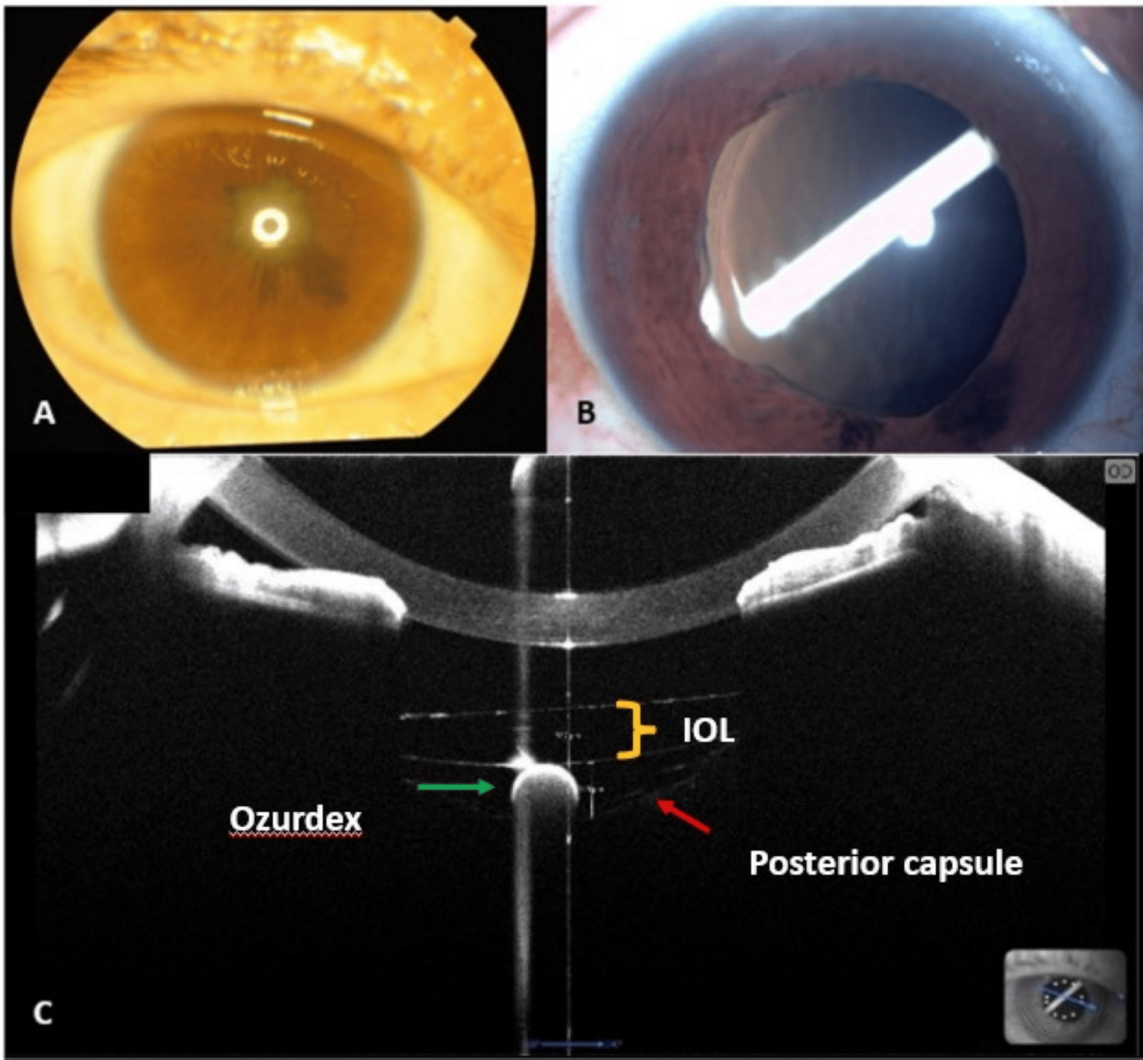
(A) Preoperative slit lamp image of the right eye showing complicated cataract with 360° posterior synechiae. (B) Postoperative slit lamp image showing the long axis of the Ozurdex® implant within the capsular bag. (C) Anterior segment optical coherence tomography showing the implant (green arrow) position within the capsular bag , between the posterior capsule (red arrow) and the posterior surface of the IOL (yellow bracket). IOL: intraocular lens

On examination, her best-corrected visual acuity (BCVA) was counting fingers at 1 m in the right eye and absent light perception in the left eye. Slit-lamp biomicroscopy of the right eye revealed a complicated cataract with 360° posterior synechiae with no cells or flare in the anterior chamber. (Figure 1A) Fundus details were not visible. B-scan ultrasonography of the right eye revealed a few scattered echoes in the vitreous cavity with an attached retina.

The patient underwent manual small incision cataract surgery with posterior chamber IOL implantation. Considering the underlying inflammatory component, a dexamethasone intravitreal implant (Ozurdex) was administered at the end of surgery by the anterior segment surgeon. On postoperative day one, BCVA improved to 6/36 in the right eye. The cornea was clear, with mild anterior chamber inflammation, and the IOL was well centered. Unexpectedly, the long axis of the Ozurdex implant was visualized within the capsular bag (Figure 1B). Anterior segment optical coherence tomography (AS-OCT) confirmed the implant’s position within the capsular bag, between the posterior capsule and the posterior surface of the IOL (Figure 1C). Fundus examination revealed an attached retina, and intraocular pressure remained within normal limits. The patient was managed conservatively with regular follow-up. No further complications were noted.

## Discussion

The Ozurdex implant is a biodegradable, sustained-release system that delivers dexamethasone to the vitreous cavity over several months, providing effective control of intraocular inflammation [1,3]. The implant is delivered into the vitreous cavity via a 22-gauge needle, with the applicator held parallel to the limbus and a shelved scleral path created by engaging the sclera obliquely with the bevel up. Once the needle tip is ~1 mm into the sclera, reposition it to advance toward the center of the eye, then fully depress the actuator until a click is heard before withdrawing the needle and applicator [4]. However, implant migration to unintended locations, such as the anterior chamber, sulcus, or subconjunctival space, has been documented, particularly in aphakic or pseudophakic eyes with posterior capsular compromise [2]. In the present case, the implant was noted entirely within the capsular bag despite an intact posterior capsule, an occurrence that, to our knowledge, has not been previously reported. Possible explanations include inadvertent entry of the injector tip into the capsular bag through a micro-capsular defect during injection or an anteriorly directed trajectory leading to deposition within the bag rather than the vitreous cavity. Since the implant remained stable and did not cause intraocular pressure elevation, inflammation, or optical disturbance, a conservative approach with close observation was chosen. The patient remained stable during follow-up.

## Conclusions

We report a rare occurrence of an Ozurdex implant located within the capsular bag following cataract surgery. This case underscores the need for meticulous attention to injection technique and spatial orientation during implant administration.
